# *BRCA1/2* variants and copy number alterations status in non familial triple negative breast cancer and high grade serous ovarian cancer

**DOI:** 10.1186/s13053-022-00236-y

**Published:** 2022-08-19

**Authors:** Fatima Zahra El Ansari, Farah Jouali, Rim Fekkak, Joaira Bakkach, Naima Ghailani Nourouti, Amina Barakat, Mohcine Bennani Mechita, Jamal Fekkak

**Affiliations:** 1 Biomedical Genomics and Oncogenetics Research Laboratory, Faculty of Science and Techniques of Tangier, Abdelmalek Essaadi University, Tetouan, Morocco; 2 Molecular Biology Department, ANOUAL Laboratory, Casablanca, Morocco

**Keywords:** Triple negative breast cancer, High grade serous ovarian Cancer, *BRCA1/2*, Morocco

## Abstract

**Background:**

While the role of *BRCA1/2* genes in familial breast and ovarian cancer is well established, their implication in the sporadic form of both cancers is still controversial. With the development of poly (ADP-ribose) polymerase (PARP) inhibitors, the exact relationship between *BRCA1/2* genes and sporadic triple negative breast cancer/high grade serous carcinoma (TNBC/HGSC) needs to be further investigated. Therefore, we conducted a study in which we analyze *BRCA1/2* point mutations and copy number alterations in Moroccan patients suffering from TNBC/HGSC.

**Methods:**

To achieve our goal, we analyzed *BRCA1/2* genes in the FFPE tissue blocks and blood samples of 65 TNBC/HGSC selected patients, using next generation sequencing technology.

**Results:**

From the 65 successfully sequenced patients in our cohort, we detected five-point variants in six different patients, four variants were classified as pathogenic and one of unknown significance. Regarding copy number alterations we detected one copy number loss in *BRCA1* gene and one copy number gain in *BRCA2* gene. The genetic screening of *BRCA1/2* genes using these patients’ genomic DNA indicated that five harbored a germline genetic alteration. While three harbored a somatic genetic alteration. To the best of our knowledge, three-point variants detected in our study have never been reported before.

**Conclusion:**

According to the results found in the present study, in a population without a family history of cancer, the possibility of a *BRCA1/2* somatic pathogenic variant in high grade serous carcinoma is 7%. While for Triple negative breast cancer somatic point variants and copy number alterations seems to be a very rare genetic event.

## Background

*BRCA1* and *BRCA2* genes encode for tumor suppressor proteins that are implicated in the repair of DNA double-strand breaks (DSBs). Both proteins work in a common pathway known as the DNA homologous recombination repair pathway (HRR). A pathogenic genetic alteration in one of these genes induces homologue recombination deficiency (HRD), which increases the possibility of genetic alterations and genome instability, resulting in the formation of an undetectable cancer cell [1, 2].

In this regard, numerous molecular oncology studies have been conducted over the last decade to investigate the impact of *BRCA1/2* genetic alterations and their role in cancer. Both genes have proven to play a significant role in hereditary breast and ovarian cancer [3]. Carriers of a genetic alteration in one of these genes face a 40–70% lifetime risk of developing breast cancer and 10–45% lifetime risk of developing ovarian cancer [4, 5].

While the implication of *BRCA1*/*2* genes in familial breast and ovarian cancer is widely understood, their role in the sporadic form of both cancers is still controversial. Many studies around the world have indicated the presence of an association between *BRCA1/2* genetic alterations and the sporadic form of triple-negative breast cancer (TNBC) and high-grade serous ovarian carcinomas (HGSC). Since one-third of *BRCA1/2* mutated BC patients suffer from TNBC subtype, and nearly half of OC patients with *BRCA1/2* mutations develop a HGSC subtype [6, 7]. These findings have prompted scientific researchers to speculate about the presence of *BRCA1/2* somatic genetic alterations in both cancer subtypes [8, 9].

Currently, with the introduction of poly (ADP-ribose) polymerase (PARP) inhibitors, for *BRCA1/2* mutated breast and ovarian cancer patients [4], the exact relationship between *BRCA1/2* genes and sporadic TNBC/HGSC needs to be further investigated.

As such, we conducted a study in which we analyze *BRCA1/2* point variants and copy number alterations (CNA) in TNBC and HGSC tumors. The major purpose of our study was to determine the prevalence of *BRCA1/2* somatic point mutations and CNA in both malignancies and whether they could represent a significant molecular marker.

## Material

### Study population

In this study, we enrolled 65 female patients. Thirty-seven suffered from TNBC and twenty-eight suffered from HGSC. The recruited patients were selected according to the following criteria:

Inclusion criteria:

- TNBC or HGSC.
- Age ranging from 45 to 70 years old.

Exclusion criteria:

- Family history of cancer.
- Age of diagnosis less than 45 years of age.
- Bilateral tumors.

Formalin-fixed paraffin-embedded (FFPE) tissue blocks and blood samples were collected from all the recruited patients after signing informed consent. Clinical and histopathological data were gathered by reviewing patients’ medical records. This study was approved by the local research ethics committee.

## Methods

### DNA extraction

The DNA extraction from paraffin tissue blocks and blood samples was performed automatically on the Maxwell 16 instrument, using the Maxwell 16 FFPE Tissue LEV DNA Purification Kit for FFPE samples and the Maxwell 16 Blood DNA Purification Kit for blood samples. DNA extractions were carried out in accordance with the manufacturer’s instructions.

The eluted DNA concentrations were determined using a Qubit 3.0 Fluorometer (Thermo Fisher Scientific).

### Second-generation sequencing

Genetic analysis of *BRCA1* and *BRCA2* genes (accession numbers NM_007294 and NM_000059, respectively) mutations and copy number alterations was processed using the Oncomine *BRCA1/2* research assay panel customized by Thermo Fisher. This panel contains 275 primer pairs in two pools, allowing for a comprehensive sequencing of *BRCA1/2* genes.

Briefly, library preparation was performed automatically on the Ion Chef platform using the Ion AmpliSeq Chef Solutions DL8 Kit. This was followed by clonal amplification, which was also performed automatically on the ION Chef Platform using the Ion AmpliSeq IC 200 Kit (Thermo Fisher Scientific). Finally, the prepared sequencing templates were sequenced on the Ion Personal Genome Machine System PGM (Ion Torrent; Thermo Fisher Scientific, Inc.) using Ion 318™ Chips and the Ion PGM™ Sequencing Hi-Q Kit v2, according to the manufacturer’s guidelines.

### Bioinformatics analysis

After sequencing, the generated DATA were initially processed on the Ion Torrent Suite software v5.4 (Ion Torrent; Thermo Fisher Scientific, Inc.), to generate filtered sequence reads and remove poor signal-profile reads. The resulting reads were then aligned to the H19 (GRCh37) genome, using the reference genome sequence that targets *BRCA1/2* genes. Finally, depth analysis and single nucleotide variants calling were performed using the Ion Torrent coverage and Variant Caller plug-in (version 5; Thermo Fisher Scientific).

The resulting BAM files were then analyzed on the Ion Reporter Analysis software (v. 5.10), using the Oncomine BRCA (5.10) pipeline (Thermo Fisher Scientific), which covers single nucleotide variants and copy number variations.

All the detected variants in our study were classified following the American College of Medical Genetics and Genomics (ACMG) guidelines.

## Results

All the clinical and histopathological data of our patients are summarized in Table 1. Among the 65 recruited females in our cohort, thirty-seven suffered from TNBC, whereas twenty-eight suffered from HGSC.

**Table 1:** Clinical and pathologic characteristics our selected patients

TNBC patients Characteristic	Number of patients	EOC patients Characteristic	Number of patients
Tumor Localization		**Tumor Localization**	
Left	22	**Left**	13
Right	15	**Right**	15
T-stage		**Figo-Stage**	
T1	–	**I-II**	2
T2	7	**III**	15
T3 - T4	30	**IV**	11
Lymph node status		**Lymph node status**	
Positive	18	**Positive**	18
Negative	19	**Negative**	10
Histological type			
Ductal carcinoma	27		
Lobular carcinoma	10		

The median age of the thirty-seven TNBC patients was 53 years old, ranging from 45 to 67 years old. Twenty-two (59.4%) had left-sided breast cancer and fifteen (40.5%) had right-sided breast cancer. The majority of these patients were diagnosed with invasive ductal carcinoma (27 patients,73%), while only Ten (27%) had lobular carcinoma. Regarding tumor stage, thirty patients (81%) presented with T3-T4 stage and seven (19%) were diagnosed with T2 stage. Finally, eighteen (48.6%) of our patients exhibited tumors with positive lymph nodes status and nineteen (51.3%) were lymph node negative.

As for our twenty-eight HGSC patients their median age was 56 years old, ranging from 45 to 69 years old. Fifteen (53.5%) had right ovary tumor localization, whereas thirteen (46.4%) had left ovary tumor localization. In terms of tumor stage, more than half of our patients (15 patients, 53.5%) presented with stage III, followed by stage IV (11 patients 39.2%) and lastly stage I-II (2 patients 7.1%). Finally,18 (64.2%) of our HGSC patients had a positive lymph node status while 10 (35.7%) had a negative lymph node status.

After *BRCA1/2* molecular analysis, we detected five variants in sex different patients and two copy number alterations in two different patients, all summarized in Table 2.

**Table 2:** *BRCA1/2* sequencing results

	Alteration type	Gene	Variant Name	Zygosity	Quality score	Variant Effect	Cancer Type	Germline /Somatic
**Patient 1**	**SNV**	*BRCA1*	c.66_67delAG (p.Glu23fs)	heterozygous	842	Frame-shift	TNBC and HGSC	Germline
**Patient 1**	**SNV**	*BRCA1*	c.66_67delAG (p.Glu23fs)	Heterozygous/ Homozygote	1089	Frame-shift	TNBC and HGSC	Germline
**Patient 3**	**SNV**	*BRCA1*	c.2494_2495delCCinsTT (p.Pro832Leu)	heterozygous	2018	In Frame	TNBC	Germline
**Patient 4**	**SNV**	*BRCA1*	c.4412delG (p.Gly1471fs)	heterozygous	318	Frame-shift	HGSC	Somatic
**Patient 5**	**SNV**	*BRCA2*	c.643delG (p.Glu215Lys)	heterozygous	993	Frame-shift	TNBC	Germline
**Patient 6**	**SNV**	*BRCA2*	c.7632_7633delCG (p.Val2545Phe)	homozygous	1984	Frame-shift	HGSC	Somatic
**Patient 7**	**CNV**	*BRCA2*	13q13.1(32930545–32,930,833)×3	–	36	Large Duplication	HGSC	Somatic
**Patient 8**	**CNV**	*BRCA1*	17q21.31(41249158–41,251,946)×1	–	48	Large Deletion	TNBC	Germline

c.66_67delAG (p.Glu23fs),variant was detected in the FFPE and blood samples of two different patients. The first patient was a 47 year old female suffering from TNBC, the variant was detected in both the FFPE and blood samples of the patient in a heterozygote state, with a depth score of 842 and 1150 respectively. While the second patient was 52 year old female suffering from HGSC the variant was detected in a homozygote state in the FFPE sample with a depth score of 524 and in the genomic DNA in a heterozygote state with a depth score of 1280.

c.2494_2495delCCinsTT (p.Pro832Leu), variant was detected in a heterozygote state in both the FFPE and blood samples of a 52 year old TNBC patient. In the FFPE sample this variant was detected with a depth score of 253 and in the genomic DNA it was detected with a depth score of 856.

c.4412delG (p.Gly1471fs), variant was detected only in the FFPE sample of a 56 year old HGSC patient. The patient’s FFPE sample sequencing revealed the presence of this variant in a heterozygote state with a depth score of 318.

c.643delG (p.Glu215Lysfs), variant was detected in a heterozygote state in both the FFPE and blood samples of a 49 year old TNBC patient. The patient’s sequencing results revealed the presence of this variant in the FFPE sample with a depth score of 289 and in the genomic DNA sample with a depth score of 993.

c.7632_7633delCG (p.Val2545Phefs), variant was detected in the FFPE sample of a 58 year old HGSC patient. The patient FFPE sample sequencing revealed the presence of this variant in a homozygote state with a depth score of 1984. This variant was not detected in the patient’s genomic DNA.

The first copy number variation detected in our study is a copy number loss 17q21.31(41249158–41,251,946)× 1 in *BRCA1* gene. This CNV was detected in the FFPE and blood samples of a 52 year old TNBC patient, with a confident score of 45 and 38 respectively.

The second copy number alteration detected in our study is a copy number gain, 13q13.1(32930545–32,930,833)× 3 in *BRCA2* gene. This CNA was detected in the FFPE sample of a 55 year old HGSC patient, with a CNV confidence score of 36, and it was confirmed by another sequencing run, in which it was also detected with a CNV confidence of 32. This copy number gain was not detected in the patient’s genomic DNA.

## Discussion

We conducted a study in which we analyzed *BRCA1/2* point variants and copy number alterations in 37 TNBC and 28 HGSC patients. The main goal of our study was to determine the prevalence of *BRCA1/2* somatic variants and copy number alterations in both malignancies and whether they could represent a significant molecular marker.

Of the 65 successfully sequenced patients in our cohort, we were able to identify five-point variants in six different patients, three in *BRCA1* gene and two in *BRCA2* gene. Regarding copy number alterations we detected one copy number loss in *BRCA1* gene and one copy number gain in *BRCA2* gene. The genetic screening of *BRCA1/2* genes using these patients genomic DNA indicated that five harbored a germline *BRCA1/2* genetic alteration. While three harbored a somatic *BRCA1/2* genetic alteration (Table 3).

**Table 3:** Characteristics of the detected *BRCA1/2* genetic alterations

Variant	Patients number	Length (pb)	Exon	Variant pathogenicity	Reference
c.66_67delAG (p.Glu23fs)	2	2	Exon 2	Pathogenic	Clinvar
c.2494_2495delCCinsTT (p.Pro832Leu)	1	2	Exon 10	Uncertain significance	Current study
c.4412delG (p.Gly1471fs)	1	1	Exon 14	Pathogenic	Clinvar
c.643delG (p.Glu215Lys)	1	1	Exon 8	Pathogenic	Current study
c.7632_7633delCG (p.Val2545Phe)	1	2	Exon 16	Pathogenic	Current study
13q13.1(32930545–32,930,833)×3	1	288	Exon 15	Uncertain significance	Current study
17q21.31(41249158–41,251,946)×1	1	2788	Exon 7–8	Pathogenic	Current study

The first variant reported in our study is c.66_67delAG (p.Glu23fs). It was detected in two different patients one was diagnosed with TNBC and the second with HGSC. According to Clinvar database, c.66_67delAG (p.Glu23fs) is a pathogenic variant of *BRCA1* gene, caused by the deletion of two nucleotides, Adenine and Guanine at exon 2 of the gene. At the protein level, this deletion provokes the substitution of Glutamic acid by Valine at codon 23, which leads to a premature stop codon at position 16 of the new reading frame [10]. This pathogenic variant was reported by Struewing et al. as a founder mutation of the Ashkenazi Jewish population [11]. Also, it has been reported in many other populations worldwide and in the Moroccan population as well [12, 13].

The second variant reported in our study is c.2494_2495delCCinsTT (p.Pro832Leu). It was detected in both the genomic and the FFPE DNA of a TNBC patient. This variant is due to the deletion of two cytosine nucleotides and the insertion of two thymine nucleotides at exon 10 of *BRCA1* gene. At the protein level, this variant is expressed by the substitution of proline amino acid by leucine. To the best of our knowledge, this variant has never been reported before in any database or research study.

The third variant reported in our study is c.4412delG (p.Gly1471fs). It was detected only in the FFPE DNA of a HGSC patient. According to Clinvar database, this variant is a pathogenic mutation of *BRCA1* gene that results from the deletion of a guanine nucleotide at exon 14 of *BRCA1* gene. At the protein level, it causes the substitution of a Glycine amino acid by Alanine at codon 1471 which introduces a premature stop codon at position 34 of the new reading frame. This variant was reported before by Winter et al. as a somatic mutation in a breast cancer case [14].

The fourth variant reported in our study is c.643delG (p.Glu215Lys). It was detected in both the genomic and the FFPE DNA of a TNBC patient. This variant is due to the deletion of guanine nucleotide at exon 8 of *BRCA2* gene. At the protein level, it causes the substitution of glutamic acid by lysine, which creates a premature stop codon at position 658 of the new reading frame. To the best of our knowledge, this variant is not reported in any database or research study.

The last variant reported in our study is c.7632_7633delCG. It was detected only in the FFPE DNA of a HGSC patient. This variant is due to the deletion of two nucleotides; guanine and cytosine at exon 16 of *BRCA2* gene. At the protein level, it causes the substitution of valine amino acid by phenylalanine at position 2545, which provokes a premature stop codon at position 2547 of the new reading frame. To the best of our knowledge, this variant has never been reported before.

Regarding copy number alterations, we detected one copy number loss in *BRCA1* gene 17q21.31(41249158–41,251,946)× 1, The latter was detected in both the genomic and the FFPE DNA of the patient. *BRCA1* gene exon deletions have been reported before in HBOC cases [15, 16]. Moreover, Pan et al., have reported *BRCA1* gene exon 7–8 deletion, in hereditary ovarian cancer cases [17].

We also detected one copy number gain 13q13.1(32930545–32,930,833)× 3 in *BRCA2* gene. This copy number alteration was not detected in the patient’s genomic DNA. *BRCA2* exon 1–2 copy number duplications have been reported before in HBOC cases [18]. However, exon 15 somatic duplication in breast or ovarian cancer has not been reported before in any database or research study.

In our study, from the 37 successfully sequenced TNBC patients, we didn’t detect any somatic point variants or copy number alterations in both genes. Our study results line up with those reported by several research studies worldwide [19, 20] Nevertheless, other research studies reported somatic point variants in non-familial TNBC cases with a prevalence between 3 to 5% [21, 22]. On the other hand, from the 28 successfully sequenced HGSC patients, we detected two somatic pathogenic variants in *BRCA2* gene (7%) and one somatic copy number gain also in *BRCA2* gene (3.5%). Our findings are consistent with most studies worldwide, which reported *BRCA1/2* somatic pathogenic variants in non-familial HGSC, with a prevalence of 5 to 8% [23–25]. However, concerning copy number alterations as far as we know most studies worldwide perform a comprehensive CNA analysis in non-familial TNBC and HGSC cases. Unfortunately, we couldn’t find a study that reported *BRCA2* somatic copy number gain in HGSC [26, 27].

In our cohort four of our patients out of 65 (6%) harbored a *BRCA1/2* pathogenic germline alteration, we concluded that these cases suffer from hereditary breast and ovarian cancer. Even though our selection criteria aimed to avoid HBOC patients. Several studies worldwide reported variable frequencies of *BRCA1/2* germline variants in unselected TNBC and HGSC cases [28, 29]. All four patients that harbored *BRCA1/2* germline alterations in our cohort confirmed the absence of cancer history in their families. Unfortunately, we could not analyze the genetic profile of their descendent parents. As a result, we could not confirm the hereditary origin of these genetic alterations. As far as we are concerned, they could be de novo alterations, which has been reported before by Kim De Leeneer et al. [30]. Moreover, we cannot neglect the fact that a respectable percentage of breast and ovarian cancer patients, with inherited *BRCA1/2* genetic alterations, do not have a clear family history, due to a small family structure, the predominance of males in the family, and paternal inheritance [24]. This can only indicate that *BRCA1/2* genetic testing should be considered for TNBC and HGSC patients even with the absence of hereditary breast and ovarian cancer selection criteria.

In the present study, we detected three point variants and one copy number gain that has never been reported before; c.2494_2495delCCinsTT (p.Pro832Leu), c.643delG (p.Glu215Lys), c.7632_7633delCG and 13q13.1(32930545–32,930,833)× 3. The Oncomine variant class pipeline has classified both variants c.643delG (p.Glu215Lys) and c.7632_7633delCG as truncating variants that causes a loss of normal protein function. While c.2494_2495delCCinsTT (p.Pro832Leu) variant was not classified by the Oncomine variant class pipeline therefore, to determine the pathogenicity of this variant we used the in silico analysis results given by SIFT (TI = 0.36), PolyPhen (PISC = 0.07) and GRANTHAM (R = 98). These results demonstrate that this variant is still of unknow significance. Apropos the copy number gain 13q13.1(32930545–32,930,833)× 3 the latter was also not classified by the Oncomine pipeline. However, since *BRCA2* gene is a tumor suppressor gene. Copy number gains are not regarded as a cancer driver genetic alterations [28]. Nonetheless, they are considered as up-regulating genetic alterations that, depending on their position in the gene, may cause a protein overexpression. BRCA2 overexpression has been reported before in research studies that analyzed the expression profile in breast cancer tumors [31, 32]. More studies should be conducted regarding *BRCA1/2* copy number variation and their phenotypical effects.

Both variants c.643delG (p.Glu215Lys) and c.66_67delAG (p.Glu23fs). Were detected in a homozygote state in the patient’s FFPE DNA. The second variant was detected in the patient genomic DNA as well in a heterozygote state. The fact that the latter is a germline pathogenic variant that was present in the patient’s FFPE DNA in a homozygote state, encourages us to support the two genetic hit theory proposed by Knudson et al. [33]. Nevertheless, three other patients in our cohort presented a germline *BRCA1/2* pathogenic variant and they didn’t present a loss of heterozygosis or compound mutations in *BRCA1/2* genes in their FFPE DNA. The genetic cancerogenesis process of hereditary breast and ovarian cancer is still not well established but many scientific studies have indicated that these tumors need an additional somatic mutation in other tumor suppress genes or oncogenes [34, 35].

To the best of our knowledge, this is the first study to analyze *BRCA1/2* point variants and copy number alterations in the FFPE DNA of Moroccan TNBC and HGSC patients. Effectively, we were able to report two somatic point variants and one somatic copy number gain in HGSC patients. Moreover, we were able to identify three germline point variants that to the best of our knowledge were never reported before. This study offers information of clinical importance regarding *BRCA1/2* genetic screening in non-familial cases of TNBC and HGSC. Nonetheless, the small number of cases in our cohort and the absence of information concerning the methylation profile of both genes are two limitations of our study, that should be surpassed in other future Moroccan studies dedicated to the implication of *BRCA1/2* genes in breast and ovarian cancer.

## Conclusion

According to the results found in the present study, the possibility of a *BRCA1/2* somatic pathogenic mutation in HGSC is 7%. While in the case of TNBC, point mutations and CNA seems to be a very rare genetic event. Nevertheless, many studies worldwide have indicated the presence of somatic *BRCA1/2* genetic alterations in TNBC. Moreover, in our cohort 6% of our TNBC/HGSC cases harbored a germline genetic alteration in *BRCA1/2* genes which indicate that a respectable percentage of non-familial breast and ovarian cancer may harbor a *BRCA* pathogenetic germline alteration. With the emergence of Anti-PARP treatment, defining the extant of *BRCA1/2* genes implications in non-familial BC and OC has become inevitable. Therefore, more studies should be conducted to define the intensity of both genes’ implication in these subtypes of cancer.

## Data Availability

The datasets analyzed during the current study and a list of material requirement will be available from the corresponding author on reasonable request.

## Abbreviations

TNBC: Triple negative breast cancer
HGSC: High-grade epithelial ovarian cancer
BC: Breast cancer
OV: Ovarian cancer
CNV: Copy Number Variation
FFPE: Formalin-fixed paraffin-embedded
PARP: Poly (ADP-ribose) polymerase
DSBs: Double-Strand Breaks
HRR: Homologous Recombination Repair
HRD: Homologous Recombination Deficiency
CNA: Copy Number Alteration
HER-2: Human Epidermal Growth Factor Receptor 2
PGM: Personal Genome Machine
